# Equity focused workshops: Developing a supplementary method to judge the equity of health interventions

**DOI:** 10.1080/29944694.2025.2530969

**Published:** 2025-07-14

**Authors:** Gemma Spiers, Jane McDermott, Eugenie Evelyn Johnson, Tafadzwa Patience Kunonga, Barbara Hanratty

**Affiliations:** a National Institute for Health and Care Research (NIHR) Policy Research Unit in Healthy Ageing, Population Health Sciences Institute, Newcastle University, Newcastle-upon-Tyne, UK; b National Institute for Health and Care Research (NIHR) Policy Research Unit in Healthy Ageing, School of Health Sciences, Faculty of Biology, Medicine and Health, The University of Manchester, Manchester, UK

**Keywords:** Equity, methods, disadvantage, socioeconomic status

## Abstract

Systematic reviews that consider the equity of interventions are an important method. However, this approach can be limited when analyses of inequity for a specific intervention are rare. We aimed to develop and pilot Equity-Focused Workshops (EFWs), a method to supplement evidence synthesis approaches when primary studies do not report data about intervention equity. A workshop protocol was developed with methods experts, focusing on understanding equitable access to interventions between socioeconomic groups. We tested the protocol in three workshops with populations experiencing material disadvantage, using two hypothetical interventions. Data were collected about participants’ views of the intervention, including acceptability and accessibility. Data were summarised and reviewed to appraise the utility of the method to judge intervention equity between socioeconomic groups. Seventeen participants discussed numerous reasons why they might find it difficult to engage with the interventions. These barriers were material, non-material, and offered insight into the ways in which interventions may exclude disadvantaged populations. Issues of stigma, professionals’ understanding of material difficulties, and the costs incurred by participants using the interventions were highlighted. EFWs can offer preliminary insights into whether health interventions are likely to exclude populations experiencing material disadvantage. There is value in this approach to supplement evidence syntheses.

## Background

Equity in health means that opportunities to stay healthy are fair across populations. In an equitable health system, access to care is equal for populations with equal needs (Whitehead, 1991). When interventions are not equitable, they risk widening inequalities in health. A critical component of applied health research is therefore to assess the equity of interventions.

Equity-focused systematic review (EFSR) is a method that aims to assess whether health interventions produce equal benefits across populations (Tugwell et al., 2010; Welch et al., 2016). This is an important instrument in health research, helping to identify the positive or negative impact of interventions on health inequalities. EFSRs require primary studies to report the outcomes of interventions by characteristics such as gender or socioeconomic status. However, evaluations of health interventions rarely report these data (Kunonga et al., 2023). When such data are not reported in the primary studies included within a review, researchers are unable to draw clear conclusions about the equity impact of a health intervention.

There are many options available to researchers to evaluate how equitable the impacts of health interventions have been. Economic evaluations use a range of methods to consider whether interventions are likely to offer equitable health outcomes across populations (Cookson et al., 2009). Qualitative and realist methods can reveal important insights about barriers to engaging with interventions that may exclude some groups from the intended benefits (Green & Thorogood, 2018; Pawson & Tilly, 1997). Stratification of evaluation outcomes by population subgroups, or consideration of demographic variables as moderators of treatment effects, can show whether interventions favour or marginalise certain groups. Despite these options, it is not always feasible for evaluations to incorporate such methods. Available resources may pose limits on what is achievable (Meadmore et al., 2023). Critically, the population subgroups, for whom equity could be assessed, are wide ranging and evaluators will need to prioritise equity appraisals for some groups over others (Cookson et al., 2009).

In the health sciences, we are seeing greater importance placed on equity assessments within evaluations of health interventions. One example of this is the development of For Equity, an online resource from the United Kingdom, to support researchers to incorporate equity considerations in their methods (NIHR School for Public Health Research, n.d.). A recent review also found that equity is being considered more frequently in economic evaluations (Avanceña & Prosser, 2021). Even so, progress at incorporating equity considerations into evaluations remains slow, particularly within the field of healthy ageing (Kunonga et al., 2023). The lack of data about how intervention effects vary by population sub-groups render EFSRs challenging to undertake.

One way to maximise the use and value of EFSRs is to supplement the method with additional data about the potential impact of interventions on equitable experience or outcomes of interventions. Such data could be collected from the population(s) whom the research team hypothesise may be excluded from the benefits of the intervention. These data could then be used to make preliminary judgements about the equity of an intervention(s) identified in a systematic review. This approach is unlikely to generate firm conclusions about equity. However, it may offer a useful starting point when there is no or little evidence available from the reviews’ included primary studies.

To progress this option, we aimed to develop and test Equity-Focused Workshops (EFWs) as a method to supplement EFSRs.

## Methods

For the development of this method, we focused on equity in health outcomes between socioeconomic groups. Understanding how health interventions impact on different socioeconomic groups is a critical consideration in public health policy, especially as life expectancies continue to diverge between the rich and poor (Marmot et al., 2020; Mosquera et al., 2019; Pongiglione et al., 2015; Tetzlaff et al., 2020).

A workshop approach was selected as the basis for the method for two reasons. First, we wanted to draw on the benefits of a group-based, interactive discussion to generate rich data about an intervention to improve health outcomes. This was particularly important as we aimed to focus less on participants’ past experiences, and more on the potential and anticipated barriers to a hypothetical intervention. We describe this in further detail below. Second, in line with our decision to focus on socioeconomic inequity, we aimed to recruit populations who experienced material disadvantage. Such disadvantage represents a potentially shared experience, which may lend itself well to a group discussion. This mirrors the core principals of focus group methods, where commonality of experience is exploited through group-based data collection (Parker & Tritter, 2006).

The EFW method was developed in three stages.

### Stage 1

In the first stage, we prepared a broad outline of the topics and questions for workshop participants. These topics and questions focused on a) the acceptability of, and potential barriers to, a specific health intervention, and b) anticipated incurred costs of using the intervention. These topics were drawn from the principals of process and realist evaluation (Pawson & Tilly, 1997).

As we intended to collect data from populations who experience material disadvantage, we focused specifically on barriers to accessing health interventions that may be faced by these groups. These barriers were drawn from Dalghren and Whitehead’s rainbow model of the social determinants of health (Dahlgren & Whitehead, 1991), and included material (e.g. income, digital access) and structural (e.g. transport availability) factors. We also included barriers that may stem from material or structural forms of exclusion: stigma (Reutter et al., 2009), and trust in health professionals (Loignon et al., 2015).

We shared this outline with methodological experts in health equity, including economists, implementation scientists, public health researchers and social scientists. One-to-one consultations with each expert explored our data collection intentions. We sought feedback about the feasibility of the overall approach and suggestions for data collection items, topics and workshop delivery. Using the information collected in stage 1, we developed a draft protocol for an EFW.

### Stage 2

In the second stage of this work, we undertook three workshops to pilot the protocol.

#### Participants and recruitment

A purposive sampling strategy was used to recruit older people experiencing material disadvantage. We chose to recruit older people (50+ years) to align with our broader work on healthy ageing. The threshold 50+ was used to define “older” as age-related poor health occurs at earlier points in the life course for the most disadvantaged populations (Kingston et al., 2015).

To identify older people *experiencing material disadvantage*, we used two sets of criteria. For the first workshop, eligible participants were in receipt of universal credit, pension credit or council tax support, all of which are used to support low-income populations. For the second and third workshop, we removed the above criterion and instead specified that people must have experienced difficulties paying for important items such as food, bills, and rent/mortgage in the past year.

This change was made in response to the difficulties we experienced in recruiting participants for the first workshop. However, this change in criteria offered an opportunity to compare different operationalisations of material disadvantage. By contrasting the data collected across the three workshops, we were able to judge the merits and limitations of each approach, for EFWs exploring socioeconomic inequity.

Finally, eligible participants were those living in the Newcastle, Gateshead, North Tyneside, Manchester and Salford areas, which are located in the north of England, in the United Kingdom. These locations were chosen to align with the authors’ institutions and regional networks.

Recruitment sources included foodbanks, community support groups, advertisements in shops and libraries, and online platforms. We used online and in person platforms to avoid the risk of missing digitally excluded populations.

#### Data collection

All workshops were scheduled for 1.5 hours, took place in person and followed the protocol developed in stage 1. This involved describing a health intervention, and then facilitating a discussion around views of acceptability, barriers to using the intervention, and anticipated costs. As we were piloting the data collection protocol, we discussed two health interventions in each workshop: a digital app intervention designed to promote physical activity (Shcherbina et al., 2019), and a community choir to facilitate wellbeing (Johnson et al., 2020). These interventions were drawn from recent literature, and were chosen because they a) align with current recommendations and UK policy for supporting older people’s health (National Institute of Health & Care Excellence, 2015), and b) comprise different intervention elements targeting different healthy ageing outcomes.

We took an iterative approach to data collection. That is, following each workshop, we made small modifications to questions and format as needed to optimise the discussions for the subsequent workshop. Workshop discussions were recorded and transcribed. All workshops took place between December 2023 and March 2024.

### Stage 3

In the final stage of this work, we anonymised and summarised the data collected to evaluate the EFW method. Data were summarised according to the overarching topics in the data collection protocol, with separate summaries for each workshop. This process was descriptive, rather than interpretive, and intended only to précis the workshop discussions. We reviewed the data and method to assess: a) the utility of the data to make a meaningful judgement about intervention equity; b) ways the protocol could be refined; and c) the risks and potential mitigations of the method. Our review was shared with the experts consulted in stage 1 for additional feedback. Minor changes were made to the protocol following this review and feedback, which we describe later in the paper. The final EFW protocol is provided in the supplementary materials.

#### Ethical and inclusion considerations

All participants were given information about the project, and their consent audio recorded. Data were anonymised and stored securely. Ethical approval was obtained through the Newcastle University Research Ethics Committee (ref: 34199/2023). Participants were offered a monetary voucher as a thank you for their time, and we offered to reimburse the costs of travel to and from venues where the workshops took place. Workshops took place in accessible settings, including a community centre and premises at Newcastle University.

## Findings

Seventeen older people with experience of material disadvantage participated in three workshops (Table 1). The number of participants per workshop ranged between five to six. Table 2 summarises the data collected. In the following section, we briefly summarise these data and the type of findings obtained.

**Table 1.** Participant Characteristics (*N* = 17)

Characteristics	*N*	%
*In receipt of a means-tested benefit*		
Yes	10	58.8
No	6	35.3
Unanswered/not reported	1	5.9
*Housing tenure*		
Own	7	41.2
Rent	5	29.4
Not reported	5	29.4
*Gender*		
Male	5	29.4
Female	12	70.6
*Ethnicity*		
White British	13	76.5
Other ethnicity*	4	23.5
*Anonymised due to low numbers		

**Table 2.** Summary of Data Collected at Each Stage of the EFW Protocol

Protocol component	Summary and examples of data collected	
	Physical activity app	Community choir
Material barriers	Not having a smart phone, the affordability of data and data storage, the costs of exercise clothing (the digital app for physical activity). *"Somebody on a limited income can’t necessarily afford to leave their phone gobbling data all through the day" (Workshop 2 participant)* Across workshops, material barriers to using a health app were typically discussed first before mention of non-material barriers. Participants emphasised the difficulties of affording smartphones (as opposed to non-smart mobile phones) or handsets that were good enough quality to accommodate an app, and associated data.	The costs of travel and clothing, including wearing clothing considered socially acceptable to others, the financial obligations of socialising that may result from group health activities, fees of the activity (community choir), food expenses if travelling. Across workshops, participants typically discussed non-material barriers to using the community choir first, with material barriers raised after prompting. Whilst some participant identified cost-related reasons why they would find the choir difficult to use, the emphasis of discussion (for all workshops) tended to be on barriers relating to factors other than costs.
Non-material barriers	Lack of motivation, physical and mental limitations from, and pain associated with, long-term health conditions, dislike of digital technology.	Disinterest in the choir, shyness and lacking confidence to participate in group activities, lack of motivation, compatibility with circumstances (e.g. education, caring), lack of cultural compatibility.
Health professionals’ understanding of barriers*	Views about whether health professionals would understand the material barriers to using the health app varied. Some felt that professionals would not understand or empathise, but attributed this to a lack of time during appointments or because it was not within a health professional’s remit to address these issues. Some noted that there was a lack of understanding about digital exclusion in healthcare, citing their own experiences of this. Others felt that health professionals would understand and be sympathetic.	
Role of cost-of-living challenges	Views about the acceptability of the interventions may change if the cost of living was not so expensive, but there may still be other factors that exclude people from this health activity. Even if the costs of living reduce, the benefits may not be felt enough by low-income groups to justify engaging in activities that bear costs. *“I think the cost of living sets up a vicious circle if you like because when things get tight you get rid of the things that aren’t essential [essential] and sadly, this is one of the things that would come up as not essential … it’s not food, it’s not heating, it’s not gas, it’s not the roof over your head, but it is something that helps you get through the hard times, but because it’s not an essential you are going to actually do yourself more harm by getting rid of it, but it is one of the things that’s going to go because people just can’t even afford that £2.00 sometimes.” (Workshop 2 participant)*	
Anticipated costs of using the intervention	Smartphone Data and data storage Exercise clothing	Travel Clothing/uniform Socialising Activity fees Food
Thought experiment: would removal of material/cost barriers change your view of the acceptability of the intervention?	For some participants, there was a willingness to use the app if the costs of data or the smartphone were eliminated; this was raised both spontaneously, *and* following the thought experiment. For others, non-material barriers were more important and would ultimately influence their decision to use or not use the health app. *[Referring to an example of recycling smartphones to give to others without them]: “I think that would be a great idea for us older people over 50, over 55 so that if you’ve not got a huge amount of money coming in then you can get the same stuff, but cheaper and for things like that it would be great. Because the app I think does spur you on.” (Workshop 3 participant)*	Some participants indicated that the financial barriers were not the key driver of their view of the acceptability of the choir, and felt they would at least try it. Others who were unfavourable about this activity noted that if the costs were eliminated, this would still not change their mind as the other barriers (e.g. shyness, disinterest) were more important. One noted that the financial barriers were important, but that her decision to use would be weighed against the anticipated benefits: *“I don’t go to Yoga and things like this because it costs £5.00 a time, but this is one thing where the joy of [the choir] would mitigate that and I would pay for this so, the cost is relative really to what you feel you get out of it.” (Workshop 2 participant)*In response to this question, there was some discussion of the stigma associated with accepting financial help. For example, if the financial barriers to this activity were eliminated through vouchers or financial support, this would not make the choir more acceptable. Such support may be rejected to avoid being seen as dependent. This led to a discussion about the difficulties of admitting when something was not affordable.

*This prompt was added on the advice of experts at stage 1. The purpose of this prompt was to elicit data about a) perceived stigma relating to material disadvantage, and b) whether such stigma would influence views of the acceptability of the intervention.

### Barriers to using the health intervention

We intended discussion of the health interventions to focus on material and structural barriers (e.g. income, housing, transport, stigma). Participants typically identified material barriers (e.g. relating to income and goods), rather than structural barriers (e.g. transport availability). Participants also discussed reasons why it may be difficult to engage with the proposed interventions, which were unrelated to material or structural factors (Table 2). These barriers included, for example, shyness or a dislike of digital technology.

Across all workshops, groups identified a number of material barriers for the digital app, and often these were discussed before non-material/structural barriers, and without prompting. In contrast, barriers to using the community choir did not typically relate to material circumstances; only after prompting were material barriers discussed.

### Views about whether health professionals would understand the material/structural difficulties of engaging with the intervention

Following advice from experts at stage 1, we included a prompt about whether participants felt that the material barriers that they had identified were understood by health professionals. The purpose of this prompt was to elicit data about a) perceived stigma relating to material disadvantage, and b) whether such stigma would influence views of the acceptability of the intervention.

This prompt did elicit some discussion on how health professionals may understand financial difficulties and digital exclusion of patients. It also provided useful contextual data about the material factors discussed. Issues of stigma were also highlighted in the discussion about barriers. For example, one barrier to using the community choir was not having or affording clothing that was considered socially acceptable to wear in a group setting (Table 2).

### Costs incurred

Following the discussion of material barriers, we asked participants to tell us what items they would need to pay for in order to use each of the interventions. The costs identified largely duplicated those already discussed in the earlier part of the workshop (Table 2).

### Comparing the relative importance of types of barriers to using the health intervention

The discussion of both material and non-material barriers offered the opportunity to compare the relative importance of each. In the first workshop, we recorded the barriers discussed on sticky paper notes and grouped them into material and non-material. We then asked participants which they considered most important. This approach generated discussion about what factors were most pertinent in shaping their views of whether they would choose to engage in the intervention.

In the second and third workshop, we iterated our approach to understanding the relative importance of material and non-material barriers: a thought experiment. We asked participants to imagine that the identified costs and material barriers to the health intervention were removed: would this change their view of the acceptability and ability to engage with the intervention, or would other (non-material/cost) barriers still play a role? This prompted discussion on whether the material barriers were the primary reasons for anticipating difficulty using an intervention, or secondary to other factors (see Table 2 for data examples).

## Discussion

We developed a method to gain insights into how equitable the impact of health interventions might be, across socioeconomic groups, where no data have been identified in systematic reviews to judge this. Experts across health-related disciplines advised on our proposed protocol, which we then tested across three workshops. In this section, we reflect on: whether this method reliably enables a judgement of intervention equity; potential refinements to the workshop protocol; and the risks and potential mitigations of the method.

### Does this method generate data to make a meaningful judgement about intervention equity across the most and least advantaged groups?

Our pilot of the workshop protocol indicates that this method can generate data to make a preliminary judgement on intervention equity. To guide this judgement, we recommend interrogating the data using four questions (Table 3). First, researchers must consider the type, range and role of material or structural barriers identified (question 1). Addressing this question should articulate what it is about an intervention (if anything), and the context in which it is delivered, that prevents people who experience material disadvantage from engaging with it in a meaningful way.

**Table 3.** Questions to Judge Intervention Equity from Workshop Data

Data interrogation questions to judge equity
Were material barriers to using the intervention identified, and if so, what were these barriers? In terms of importance, where did these material barriers sit relative to non-material barriers? In the thought experiment, did elimination of material barriers change views about acceptability/willingness to use? Were non-material barriers potentially exposed to social patterning with some groups likely to be excluded as a result?

Consideration of the relative importance of material and non-material barriers is also important (question 2). Understanding whether the acceptability of an intervention is driven by material or non-material reasons can feed into researchers’ judgements of whether the intervention is likely to exclude groups experiencing material disadvantage. Such insight can also be gained from the thought experiment we included in the protocol, where participants are asked to judge the weight of potential costs and material barriers in their decision making about using the intervention (question 3).

Researchers should consider the non-material/structural barriers identified, and whether these are exposed to social patterning (question 4). For example, some participants cited the physical and mental limitations associated with a long-term health condition as reasons why they would struggle to engage with the physical activity app. Poor health is more common in the most disadvantaged groups (Watt et al., 2022), indicating that these populations may be systemically excluded from engaging with this type of intervention.

These questions should serve as a tool from which to interrogate the data and make a judgement on intervention equity. We do not prescribe a specific or single type of qualitative analysis to support this interrogation. However, narrative or thematic approaches may be most suitable to provide a rich description of the barriers identified. We did not undertake such an analysis here, as our primary goal was to assess the utility of the data collected to evaluate the EFW method.

The EFW protocol is designed to identify the range of likely barriers to using an intervention, rather than determining which are the most important or relevant. However, researchers using EFWs may wish to relate the barriers identified to the possible contexts in which the intervention might be delivered. This might include, for example, considering what other contextual factors may mitigate or exacerbate the impact of barriers identified.

In terms of integrating EFW findings into evidence syntheses, researchers should ascertain what would work best to clearly convey their appraisals. Options include locating EFW findings alongside the results of an evidence synthesis, or reporting the EFW outputs separately as a standalone project. Existing frameworks to integrate qualitative and quantitative primary studies in reviews (e.g. Thomas et al., 2004) could also potentially be adapted to accommodate the additional data generated by EFWs.

### Refinements to the method

Following our review of the data and discussion with experts in stage 3, we made minor revisions to the protocol. These included: adding an explicit prompt to explore intersectional lenses on the identified barriers; inclusion of a comparator workshop with groups not experiencing material disadvantage to qualitatively contrast views; and including explicit reference to the mechanisms of action (of the intervention) within the discussions. In addition, the methods experts highlighted the potential interdependency of material and non-material barriers, and the importance of considering both in this method.

Finally, our pilot of the workshop suggested that a separate section on anticipated out of pocket costs largely duplicated the data generated through the discussion of barriers. We have therefore removed this from the protocol. The final workshop data collection protocol is available in the *supplementary materials*.

### Risks and mitigations of the method

A core component of the EFW method is the recruitment of populations who would hypothetically be excluded from the utilisation and potential benefits of an intervention. This is necessary to gain insights about the acceptability and barriers to the intervention in question. A key risk of this method, therefore, is failing to recruit the target populations.

We used two different sets of criteria to recruit populations experiencing material disadvantage: *receipt of means tested financial support* (workshop 1) and *has experienced difficulty paying for important items in the past year* (workshops 2 and 3). The former represents objective criteria, and the latter, subjective criteria. However, both approaches allowed us to access the relevant populations. This was evident based on participants’ descriptions of their day-to-day experiences. A portion of participants in the second and third workshops were also in receipt of means tested financial support. A comparison of the data collected in the first and subsequent workshops did not suggest any stark differences in range and content. This suggests that the two different criteria identified comparable populations.

When considering the risk associated with recruitment for this method, it is important to recognise that there is no one version of material disadvantage (Smith & Middleton, 2007). Therefore, there is no single correct way of identifying people experiencing disadvantage, or a set of criteria from which to recruit participants with this experience. We would also caution against reliance on criteria that may be considered objective. For example, take-up of means-tested benefits among eligible populations is low in the UK (Department for Work and Pensions, 2024). Objective criteria such as this may risk excluding otherwise eligible populations who can offer rich and relevant insights. The methods experts we consulted with as part of our review of data also endorsed this view.

Finally, it is likely that EFWs may be used by researchers to explore the equity of interventions developed in countries other than their own. Socio-economic and cultural differences between countries should be considered, especially if interventions are designed in response to needs specific to a particular country context.

### Implications for future research

With the growing emphasis placed on considering equity in evaluations of health interventions, we expect that future studies will report the data necessary to judge equity in systematic reviews. Until this point, we advocate the EFW method as a way to supplement EFSRs where there is little or no data in primary studies from which to judge intervention equity.

EFWs are not intended to replace other, more established, methods to judge the equity of health interventions: process evaluations, sub-group analyses in trials, and economic approaches to equity impact (Cookson et al., 2009; Green & Thorogood, 2018; Pawson & Tilly, 1997). Where possible, these methods should always form part of a robust evaluation. However, resource constraints make it difficult to incorporate some of these methods into evaluative studies. EFWs offer a useful, less time-intensive, alternative. The data collected through this approach gives a preliminary picture of the health equity risks posed by interventions, including whether health benefits may favour some groups over others. Further work using more established methods can then build on these insights. The EFW method is also conducive to fast-paced policy research such as rapid evidence syntheses.

A merit of this method is its flexibility. That is, we make no distinction as to how little equity data within a systematic review should necessitate an EFW. Researchers should use their own judgement as to whether further evidence and insight is needed on the equity of an intervention. For example, researchers may identify several studies reporting an equity analysis within a review, but where data quality is poor. In such case, use of an EFW may help provide useful context from which to interpret the review findings. While PRISMA-E offers clear guidance on reporting equity findings, EFWs can add valuable insights by directly involving groups who might otherwise be overlooked, providing a deeper understanding of equity issues.

Finally, applications of this method will incur costs, particularly to ensure workshops are inclusive. Participant remunerations, travel costs, venue hire, and researcher time are required for EFWs. Such costs may not be feasible for teams with limited resources. However, the approach is flexible, and where needed, pragmatic adaptations can be applied (such as holding fewer workshops).

### Strengths and limitations

This study offers a novel approach to understanding health equity. The development of this method was informed by a broad range of insights, including methodological experts and people with experience of material disadvantage. These diverse insights, used to develop and pilot the workshop protocol, is a key strength of our study. Our recruitment strategy did not depend on digital platforms, and thus the risk of digital exclusion was low. This was critical for the focus of our work.

A limitation of our approach was that we focused on one dimension of equity: material disadvantage. Inevitably, this may exclude intersectional considerations. However, our approach to data collection offered flexibility for participants to relate aspects of their experience beyond material disadvantage. For example, in one workshop there was discussion of the intersection between material and cultural barriers to using interventions. We have also added an explicit prompt in the workshop protocol to explore intersectionality on the barriers discussed by participants. Going forward, future developments of this method may wish to give greater consideration to these intersectional dimensions of disadvantage. Adaptations of the protocol to address other dimensions of equity (such as between gender, or rural/urban populations (O’Neill et al., 2014)) are also encouraged. Researchers wishing to adapt the protocol should follow a similar approach: collecting data about acceptability and accessibility from the populations they hypothesise may be excluded from the benefits of an intervention. The underlying workshop structure may remain the same, with adaptations to the data collection questions and prompts.

## Conclusion

EFWs are a method that allow researchers to gain a preliminary insight into whether health interventions are likely to exclude populations experiencing material disadvantage. There is value in this approach to supplement EFSRs, evaluations, and rapid policy research. Going forward, future developments of this method should consider other dimensions of disadvantage.

## Supplementary Material

EFW Paper Supplementary Materials 280624.docx
