# Supplementary material for: Equity focused workshops: Developing a supplementary method to judge the equity of health interventions
Source: J Health Equity. 2025 Jul 14;2(1):2530969. doi: 10.1080/29944694.2025.2530969 (PMC12315834; doi:10.1080/29944694.2025.2530969)
Supplement: EFW Paper Supplementary Materials 280624.docx [file TJHE_A_2530969_SM6771.docx]

Supplementary Materials

Draft Equity Focused Workshop Protocol (used for piloting)

**Present the health intervention:**

Referral/access routes

Intervention objective

Steps/procedures

Expectations/requirements

**Views of acceptability and potential material barriers to using health intervention**

E.g. material barriers to prompt: the area where you live, quality/availability of community/green space, housing space, housing stability, costs of e.g. travelling, equipment needed such as clothing, digital technology/data, time away from work/work precarity/discrimination against health; trust, stigma, peer pressure, stressors related to material barriers (potentially relate to previous experiences).

Review barriers noted on flip chart.

How important are each of these barrier/ how likely would they prevent you from using this [/xxx]?

If a health professional (e.g. GP, nurse) asked you to use this [intervention], how important is it that they understand these sorts of challenges in order for you to consider using it?

Macro-context: Prompt about cost-of-living context in relation to any barriers raised/identified.

**Anticipated costs**

*What sort of items do you think you might need to pay for in order to use this [xxx]?*

Prompts: buying clothing/equipment, digital data, travelling (e.g. bus fare, petrol), covering the costs of child care or other caring, time away from work

If these costs were eliminated, would this change your view of whether you would use the [intervention]?

**Workshop data collection**

**Overall likelihood of using intervention?**

**How do you think your health would change if using this intervention?**

Finalised Equity Focused Workshop Protocol

**Objective: To collect data that will enable a preliminary judgement about whether a health intervention might a) exclude people experiencing material disadvantage, and b) deliver (in)equitable outcomes across rich and poor populations.**

*Workshop participants: populations experiencing material disadvantage (see paper for potential criteria); optional: comparator groups to explore and contrast findings across populations.*

- ***receive only the state pension as income/do not receive a private pension (if above the state pension age)***

| **Data Collection** |
| --- |
| ***Part 1*** |
| **Present the health intervention:**  Referral/access routes, intervention objective(s) including the health outcomes it is intended to change, steps/procedures and mechanisms of action, expectations/ requirements of those partaking in intervention.  Clarify any questions from workshop participants about the intervention. |
| ***Part 2*** |
| **Discuss the acceptability and potential barriers to using health intervention:**  *Imagine your doctor or nurse or another health professional suggested this health activity to you. What would be your first impression?*  *What would make it difficult to engage with/take part in this activity?*  Material barriers to prompt: the area where you live, quality/availability of community/green space, housing space, housing stability, anticipated costs (e.g. travelling, equipment needed such as clothing, digital technology/data), time away from work/work precarity/discrimination against health; trust, stigma, peer pressure, stressors related to material barriers (potentially relate to previous experiences).  If participants do not discuss anticipated costs, ensure these are prompted (in order to identify anticipated out of pocket costs).  Encourage discussion of both non-material and material barriers.  Review barriers noted on a flip chart or using post it notes.  *How important are each of these barriers? How likely would they prevent you from using this [activity]?*  *If a health professional (e.g. GP, nurse) asked you to use this [activity], how important is it that they understand these sorts of challenges in order for you to consider using it?*  *To what extent do you think these challenges might change if the cost-of-living were to improve?*  Intersectional prompt: what other barriers are important (e.g. cultural), and how do these relate to the material barriers discussed? |
| ***Part 3*** |
| **Thought experiment:**  *You have identified [summarise the material/cost barriers]. If these material barriers/costs were eliminated, would this change your view of whether you would use the [intervention]? Why?*  Prompt: relative importance of material barriers compared to non-material barriers. |
